# Sexual Power and HIV Risk, South Africa^1^

**DOI:** 10.3201/eid1011.040252

**Published:** 2004-11

**Authors:** Audrey E. Pettifor, Diana M. Measham, Helen V. Rees, Nancy S. Padian

**Affiliations:** * University of the Witwatersrand, Johannesburg, South Africa;; † University of California at Berkeley, Berkeley, California, USA;; ‡ University of California at San Francisco, San Francisco, California, USA

**Keywords:** HIV, Condoms, South Africa, women, sexual behavior, power, adolescents

## Abstract

Among a sample of young women, limited sexual power was associated with inconsistent condom use but not directly with HIV.

Gender power inequities are believed to play a key role in the HIV epidemic through their effects on women's power in sexual relationships. We hypothesized that lack of sexual power, measured with a four-point relationship control scale and by a woman's experience of forced sex with her most recent partner, would decrease the likelihood of consistent condom use and increase the risk for HIV infection among sexually experienced, 15- to 24-year-old women in South Africa. While limited sexual power was not directly associated with HIV, it was associated with inconsistent condom use: women with low relationship control were 2.10 times more likely to use condoms inconsistently (95% confidence interval [CI] 1.17–3.78), and women experiencing forced sex were 5.77 times more likely to use condoms inconsistently (95% CI 1.86–17.91). Inconsistent condom use was, in turn, significantly associated with HIV infection (adjusted odds ratio 1.58, 95% CI 1.10–2.27).

In 2002, the prevalence of HIV infection among South African women attending antenatal clinics was 26.5% (*1*). Among all 15- to 24-year-olds, 12% of women were infected, compared with 6% of men (*2*). While women's greater biological susceptibility to HIV helps explain this difference, a host of sociocultural and economic factors rooted in gender power inequities exacerbate women's vulnerability to infection.

Gender power inequities play a key role in the HIV epidemic through their effects on sexual relationships (*3**–**5*). In South Africa, multiple partnerships are condoned and even encouraged for men, while women are expected to be monogamous and unquestioning of their partner's behavior (*5**–**7*). Sexual refusal or negotiation may result in suspicions of infidelity and carry the risk of violent outcomes (*8**,**9*). Younger women are likely to be at a particular disadvantage, as documented by a growing body of qualitative research (*6**,**8**,**10*). A study of youth in a Xhosa township, for example, showed "pervasive male control over almost every aspect of [women's] early sexual experiences," enacted in part through violent and coercive sexual practices (*8*).

A host of economic vulnerabilities underlies young women's inability to challenge the sexual status quo. In the context of poverty, young women speak of money as the driving force for sex and relationship formation (*9**,**11*). Partnerships with men who can provide financially are essential, transactional relationships (in which sex is exchanged for material goods or other support) are common, and relationships with older men are the norm (*12**,**13*).

Several studies in the region have found that women's status or household power has effects on general contraceptive use (*14**–**18*). Very few studies have focused on younger women, attempted to measure relationship power directly, or assessed its effects on HIV-preventive behaviors. One exploratory study in Botswana found that negotiating power explained 47% of the variance in condom use (*19*). A study in Uganda had more mixed results, finding that relative control over sex and fertility had variable effects on condom use, depending on which partner's reports were used, and whether partner reports were in conflict (*20*).

A larger body of research exists on relationship power and HIV-preventive practices in the developed world, primarily among ethnic minorities in the United States. These studies have used a range of measures in their efforts to quantify relationship power, and some have had null or inconclusive results (*21**–**23*). A few studies have documented important effects, finding that women with greater sexual relationship power are more likely to use condoms or to use condoms consistently (*24**,**25*).

We undertook a preliminary exploration of the effects of sexual power on both HIV serostatus and condom use consistency by using data collected from a nationally representative sample of sexually experienced young women, 15–24 years of age, in South Africa. While investigating sexual power was not the primary aim of the survey, a set of questions on related issues was included.

## Methods

### Sample

In 2003, data on sexual power, HIV risk behaviors, and HIV serostatus were collected during a nationally representative household survey of men and women 15–24 years of age. Participants were selected thorough stratified, disproportionate, systematic sampling in the country's nine provinces. A total of 11,904 interviews were completed, including 4,066 with sexually experienced young women, the subsample used in this analysis. Additional details on the survey's methods are described elsewhere (*26*).

Informed consent was obtained from all participants, and parental consent was obtained for those 15–17 years of age. The study was approved by the Committee for the Protection of Human Subjects, University of the Witwatersrand, Johannesburg, South Africa.

### Measurement Tools

Participants completed an interviewer-administered questionnaire that covered sociodemographic factors, HIV risk behavior, and sexual power. All questions were translated from English into Sotho, Zulu, Tswana, Xhosa, Pedi, Venda, Tsonga, and Afrikaans, and then back-translated. Participants were anonymously tested for HIV by using the Orasure Oral Specimen Collection Device (Orasure Technologies Inc, Bethlehem, PA). The specimens were tested for HIV-1/2 antibodies by using the Vironostika Uni-Form II HIV-1/2 plus O MicroElisa System (Biomerieux, Durham, NC).

### Measures

Our primary outcome variables of interest were HIV serostatus and condom use consistency. Women who always used condoms with their most recent partner in the past 12 months were categorized as consistent condom users; never or occasional use was categorized as inconsistent use.

Sexual power was measured through two factors: relationship control and recent experience of forced sex. Four questions were used to construct the relationship control scale, and these were drawn in part from the Sexual Relationship Power Scale (SRPS) (*27*), which contains 23 items in two subscales (decision-making dominance and relationship control). A pilot test of the full scale was conducted, questions were revised, and several were eliminated due to difficulties in translating concepts, lack of comprehension among pilot test participants, and space constraints in the questionnaire. Five questions remained after this process and were examined in SPSS (SPSS Inc., Chicago, IL) by using factor analysis, which verified that four of the five questions created one factor. The four questions retained, all of which required an agree or disagree response, were as follows: your partner has more control than you do in important decisions that affect your relationship; when you and your partner have an argument, your partner gets his way most of the time; your partner has more control than you do over whether or not you use condoms; your partner has more control than you do over whether or not you have sex. Reliability analysis confirmed moderate internal consistency (Cronbach's alpha 0.69). We dichotomized the four-point scale for analytic purposes, with a score of 0–2 indicating high relationship control and 3–4 indicating low control. Forced sex was measured by asking each woman if her most recent sexual partner in the past 12 months ever physically forced her to have sex (yes or no).

In addition to the sexual power variables, we examined other participant characteristics and sexual practices that have been hypothesized to effect condom use consistency and HIV status or which might confound relationships of primary interest. These variables are presented in Table 1. In particular, an index to measure condom use self-efficacy was created by using the following questions, each of which required a yes or no answer: Would you be able to use a condom every time you have sexual intercourse? Would you be able to refuse to have sex if your partner would not use a condom? Would you be able to talk about using condoms with your partner? The index had moderate internal consistency (Cronbach's alpha = 0.60).

**Table 1:** Weighted frequencies of HIV prevalence, sexual power, sociodemographic factors, and HIV risk behavior among sexually experienced women 15–24 years of age, South Africa, 2003

Characteristic	Weighted frequency (%)
HIV positive	21.1
Low relationship control with current partner	26.6
Did not always use a condom with last partner in past 12 mo	71.4
>1 Lifetime sexual partner	54.6
>1 partner in the past 12 mo	12.8
Most recent sexual partner in past 12 mo was a regular partner	98.2
Most recent partner in past 12 mo physically forced to have sex	3.8
Ever been physically forced to have sex in lifetime	9.6
Transactional sex with most recent partner in past 12 mo	1.3
Most recent partner in past 12 mo >10 y older	5.5
Age at sexual debut <14 y	7.8
Did not talk to most recent partner in past 12 mo about using condoms	20.5
Mean condom use self-efficacy (low 0, high 3)	2.36
Perceive self to be at high risk for HIV infection	38.4
Ever been tested for HIV	32.6
Know HIV status	18.9
Had sex >5 times in past month	10.0
Reported unusual vaginal discharge in the past 12 months	19.2
Ever pregnant	49.5
Age 20–24 y	64.1
Did not complete high school	72.9
Live in a rural area	47.3
Married	4.3
Religion not important in everyday life	13.7
Black African	88.4

### Analysis

The final sample was weighted to represent the distribution of young people 15–24 years of age based on the 2001 census, with a particular focus on ensuring representativeness based on sex, age, race, province, and rural or urban residence. Analyses were conducted in STATA 7.0 (STATA Corp, College Station, TX) by using *svy* methods and adjusting for sample strata, primary sampling units, and population weights.

Chi-square tests for categorical variables and *t* tests for continuous variables were conducted to test for differences in HIV serostatus and condom use consistency by sexual power, HIV risk behavior, and sociodemographic factors. Variables were selected for the logistic regression models based on both a priori hypotheses and empiric findings. We hypothesized that relationship control and forced sex would primarily be associated with HIV indirectly through their effects on condom use, but that they could also be associated indirectly with HIV infection through other mechanisms, such as higher risk sexual practices (e.g., anal sex) or elements of unprotected intercourse not captured through the condom use consistency variable. Hence, we examined both the relationship between sexual power and condom use consistency and that between sexual power and HIV status.

## Results

HIV prevalence in our sample was 21%. Most women (71%) reported inconsistent condom use, and 12.8% reported having had more than one sexual partner in the past 12 months. Almost 27% reported low relationship control, and nearly 4% reported that they had been physically forced to have sex by their most recent partner (just under 10% reported ever having been physically forced to have sex). Approximately 50% of women reported ever having been pregnant, and 19.2% reported having had an unusual vaginal discharge in the past 12 months. Almost 19% of women reported knowing their HIV status. Other information on the sociodemographic characteristics and HIV risk behaviors of the sample is presented in Table 1.

### Bivariate Analyses

No significant association was found between low relationship control and HIV infection in bivariate analyses comparing women who were HIV infected to those who were not (24.1% vs. 28.3%, p = 0.31) (Table 2). Additionally, no association was found between the woman's experience of forced sex with her most recent partner and HIV serostatus (3.6% vs. 3.9%, p = 0.82). Women who were HIV seropositive were significantly more likely to have had more than one lifetime sexual partner, to be 20–24 years of age, to have not completed high school, to be of black African race, and to be single. HIV-positive women were also significantly more likely to be inconsistent condom users (78.7% vs. 69.6%, p = 0.01). No significant associations were found between HIV and recent experience of transactional sex, having an older partner, or young age at coital debut.

**Table 2:** Weighted frequencies and results of chi-square tests for relationship control factors, HIV risk behavior, and sociodemographic factors by HIV status among sexually experienced women 15–24 years of age, South Africa, 2003^a^

Characteristic	HIV negative (78.9%)	HIV positive (21.1%)	Chi-square p value
High relationship control (score 0–2) with current partner	71.7	75.9	0.31
Low relationship control (score 3–4) with current partner	28.3	24.1	
Last partner in past 12 mo forced to have sex	3.9	3.6	0.82
Last partner in past 12 mo did not force to have sex	96.1	96.4	
Mean condom use self-efficacy score (0 low to 3 high)^b^	2.35	2.38	0.68
Transactional sex with last partner in past 12 mo	1.3	1.5	0.89
No transactional sex with last partner in past 12 mo	98.7	98.5	
Last partner in past 12 mo >10 y older	5.1	7.5	0.08
Last partner in past 12 mo <10 y older	94.9	92.5	
Did not always use a condom with last partner in past 12 mo	69.6	78.7	0.01
Always used a condom with last partner in past 12 mo	30.4	21.3	
Age at sexual debut <14 y	7.8	7.7	0.93
Age at sexual debut >14 y	92.2	92.3	
>1 lifetime sexual partners	51.1	67.7	0.03
1 lifetime sexual partner	48.9	32.4	
Sex in past month with last partner in past 12 mo <5	90.3	89.1	0.49
Sex in past month with last partner in past 12 mo >5	9.7	10.9	
Talked about condom use with last partner in past 12 mo	80.2	76.4	0.24
Did not talk about condom use with last partner in past 12 mo	19.8	23.6	
Perceive self to be at high risk for HIV infection	38.3	39.0	0.89
Perceive self to be at low or no risk for HIV infection	61.7	61.0	
Age 15–19 y	40.4	19.0	< 0.001
Age 20–24 y	59.6	81.0	
Completed high school	29.3	18.7	< 0.001
Did not complete high school	70.7	81.3	
Live in a rural area	48.6	42.6	0.14
Live in an urban area	51.4	57.4	
Race Black African	85.9	97.9	< 0.001
Other race	14.1	2.1	
Religion very important in everyday life	86.5	85.9	0.8
Religion not very important in everyday life	13.5	14.1	
Married	4.9	2.3	0.002
Single	95.1	97.7	

^a^Source: National Survey of HIV and Sexual Risk Behavior among Young People Age 15–24, South Africa, 2003. ^b^*t* test for differences between means (tested with the *lincom* command in STATA for *svymeans*).

As we had hypothesized, inconsistent condom users were significantly more likely to report low relationship control (33.4% vs. 13.5%, p < 0.001) and to have been forced to have sex by their most recent partner (5% vs. 1%, p < 0.001) when consistent condom users were compared with inconsistent condom users in bivariate analyses (Table 3). Further, inconsistent condom users were more likely to have low condom use self-efficacy, to be in relationships with older partners, to have frequent sex with their partner, not to have discussed condoms with their partner, to be married, to have experienced early sexual debut, not to have completed high school, to perceive themselves as being at high risk for HIV infection, and to be in the older age group (20–24 years).

**Table 3:** Weighted frequencies and p values for relationship control factors, HIV status and risk behavior, and sociodemographic factors by condom use consistency with most recent sexual partner in past 12 months among sexually experienced women 15–24 years of age, South Africa, 2003^a^

Characteristic	Always used condom with last partner in past 12 mo (28.6%)	Did not always use condom with last partner in past 12 mo (71.4%)	Chi-square p value
High relationship control (score 0–2) with current partner	86.5	66.6	< 0.001
Low relationship control (score 3–4) with current partner	13.5	33.4	
Last partner in past 12 mo forced to have sex	1.0	5.0	< 0.001
Last partner in past 12 mo did not force to have sex	99.0	95.0	
Mean condom use self-efficacy score (0 low–3 high)^b^	2.68	2.16	< 0.001
Transactional sex with last partner in past 12 mo	1.1	1.5	0.6
No transactional sex with last partner in past 12 mo	98.9	98.5	
Last partner in past 12 mo >10 y older	3.2	6.5	0.01
Last partner in past 12 mo <10 y older	96.8	93.5	
Age at sexual debut <14 y	4.0	8.4	0.001
Age at sexual debut >14 y	96.0	91.6	
>1 lifetime sexual partners	47.7	41.7	0.23
1 lifetime sexual partner	52.3	58.3	
Sex in past month with last partner in past 12 mo <5	96.4	87.5	< 0.001
Sex in past month with last partner in past 12 mo >5	3.6	12.5	
Talked about condom use with last partner in past 12 mo	98.0	72.1	< 0.001
Did not talk about condom use with last partner in past 12 mo	2.0	27.9	
Perceive self to be at high risk for HIV infection	30.0	43.3	< 0.001
Perceive self to be at low or no risk for HIV infection	70.0	56.7	
Age 15–19 y	54.0	33.6	< 0.001
Age 20–24 y	46.0	66.4	
Completed high school	34.5	24.1	0.01
Did not complete high school	65.5	75.9	
Live in a rural area	38.2	51.2	0.09
Live in an urban area	61.8	48.8	
Race Black African	90.9	88.6	0.23
Other race	9.1	11.4	
Religion very important in everyday life	88.7	84.4	0.12
Religion not very important in everyday life	11.3	15.6	
Married	0.6	6.5	< 0.001
Single	99.4	93.5	

^a^Source: National Survey of HIV and Sexual Risk Behavior among Young People Age 15–24, South Africa, 2003. ^b^*t* test for differences between means (tested using the *lincom* command in STATA for *svymeans*).

### Multivariate Analyses

No direct association was seen between our two sexual power measures (relationship control and forced sex) and HIV infection in the logistic regression model (Table 4). Inconsistent condom users were significantly more likely to be infected with HIV (odds ratio [OR] 1.58, 95% confidence interval [CI] 1.10–2.27). The odds of HIV infection were 2.49 times greater among women with more than one lifetime sexual partner (OR 2.49, 95% CI 1.80–3.43) than among those with one partner. Women who were older (ages 20–24 years), were single, did not complete high school, lived in an urban area, and were of Black African race were also significantly more likely to be infected with HIV.

**Table 4:** Adjusted odds ratios (AOR), 95% confidence intervals (CI), and chi-square p values for HIV infection among sexually experienced women 15–24 years of age, South Africa, 2003^a^

Characteristic	AOR (95% CI)	Chi-square p-value
Age 20–24 y (vs. 15–19 y)	2.53 (1.85–3.46)	< 0.001
Single (vs. married)	2.06 (1.14–3.71)	0.02
Did not complete high school (vs. completed high school)	2.60 (1.87–3.61)	< 0.001
Live in an urban area (vs. live in a rural area)	2.36 (1.55–3.59)	< 0.001
Black African race (vs. other race)	7.63 (3.41–17.07)	< 0.001
Did not always use a condom with last partner in past 12 mo (vs. always used a condom)	1.58 (1.10–2.27)	0.01
>1 lifetime sexual partner (vs. 1 lifetime partner)	2.49 (1.80–3.43)	< 0.001
Last partner in past 12 mo >10 y older (vs. <10 y older)	1.43 (0.88–2.32)	0.15
Age of first sex <14 y (vs. >14 y)	1.12 (0.67–1.87)	0.66
Transactional sex with last partner in past 12 mo (vs. never transactional sex with last partner)	2.03 (0.53–7.77)	0.30
Sex >5 times in past mo with last partner in past 12 mo (vs. <5 times)	1.07 (0.72–1.58)	0.73
Last partner in past 12 mo forced to have sex (vs. did not force)	0.82 (0.45–1.52)	0.55
Low relationship control (vs. high control)	1.00 (0.72–1.39)	0.99

^a^Source: National Survey of HIV and Sexual Risk Behavior among Young People Age 15–24, South Africa, 2003.

Relationship control and recent experience of forced sex were significantly associated with condom use consistency in logistic regression models (Table 5). Women who reported low relationship control were 2.10 times more likely to be inconsistent condom users (OR 2.10, 95% CI 1.17–3.78). Forced sex was found to exert particularly strong effects on inconsistent condom use: women who reported that their most recent partner forced them to have sex were 5.77 times more likely to be inconsistent condom users with that partner (OR 5.77, 95% CI 1.86–17.91). Women who reported low condom use self-efficacy were also at increased risk of inconsistent condom use: each one-point decrease in condom use self-efficacy increased the odds of inconsistent condom use by 1.86 (95% CI 1.42–2.45). The strongest predictor of inconsistent condom use was not having talked to the most recent partner about using condoms (OR 12.86, 95% CI 5.83–28.47). Married women, women who reported having frequent sex, older women (ages 20–24 years), and women who perceived themselves to be at high risk for HIV infection were also significantly more likely to report inconsistent condom use. Early coital debut, more than one lifetime sexual partner, and having an older partner were not found to be statistically significant predictors of condom use consistency.

**Table 5:** Adjusted odds ratios (AOR), 95% confidence intervals (CI), and chi-square p values for not always using a condom with most recent sexual partner in the past 12 months among sexually experienced women 15–24 years of age, South Africa, 2003^a^

Characteristic	AOR (95% CI)	Chi-square p-value
Age 20–24 y (vs. 15–19 y)	1.87 (1.32–2.66)	< 0.001
Married (vs. single)	5.43 (2.06–14.34)	0.001
Did not complete high school (vs. completed high school)	1.28 (0.87–1.88)	0.2
Live in rural area (vs. live in an urban area)	1.25 (0.76–2.06)	0.37
Other race (vs. Black African race)	1.66 (1.01–2.73)	0.04
Perceive self to be at high risk for HIV (vs. low to no risk)	1.55 (1.13–2.11)	0.006
Low relationship control (vs. high control)	2.10 (1.17–3.78)	0.013
Last partner in past 12 mo forced to have sex (vs. did not force)	5.77 (1.86–17.91)	0.002
Condom use self-efficacy (0 high to 3 low)	1.86 (1.42–2.45)	< 0.001
Did not talk to last partner in past 12 mo about using condoms (vs. did talk about using condoms)	12.86 (5.83–28.47)	< 0.001
>1 lifetime sexual partner (vs. 1 lifetime partner)	1.22 (0.72–2.06)	0.45
Last partner in past 12 mo >10 y older (vs. <10 y older)	1.11 (0.56–2.20)	0.75
Age at first sexual experience <14 y (vs. >14 y)	1.62 (0.97–2.73)	0.06
Sex >5 times in past month with last partner in past 12 mo (vs. 0 times)	2.85 (1.69–4.79)	< 0.001

^a^Source: National Survey of HIV and Sexual Risk Behavior among Young People Age 15–24, South Africa, 2003.

## Discussion

Lack of power in sexual relationships has been hypothesized to increase women's risk of HIV infection (*3**,**4**,**19**,**28**,**29*), but little research has shed rigorous light on this question. In this nationally representative survey, women reporting limited sexual power were not more likely to be infected with HIV. Sexual power was, however, associated with inconsistent condom use, which, in turn, was significantly associated with HIV infection.

We hypothesized that limited sexual power would increase a woman's risk of HIV infection, primarily by compromising her ability to use condoms. Women with low relationship control were significantly more likely to report inconsistent condom use (OR 2.10, 95% CI 1.17–3.78), which is consistent with the findings of other studies (*25**,**30*). This finding suggests that efforts to promote consistent condom use, a key element of HIV prevention, would benefit from efforts to enhance women's sexual power. Such efforts should not target women alone; rather, they should target and involve men as partners, essential stakeholders in improving women's sexual decision-making power.

Women reporting forced sex with their most recent sexual partner were also significantly less likely to report consistent condom use (OR 5.77, 95% CI 1.86–17.91). While only 4% of our sample reported that their most recent partner had physically forced them to have sex, approximately 10% of all women reported having experienced forced sex. Since many women may be reluctant to disclose this information in a household survey, this figure is likely to be an underestimate (*31*). In the context of masculine norms defined by male control over sexual decision-making and prevalent forced and coercive sex, many women do not have the right of refusal (*6**,**8**,**10**,**32*). In addition, our measure of physically forced sex captures only a narrow element of coercive or nonconsensual sex, which actually occurs on a continuum ranging from persuasion and trickery to force and rape (*6**,**31*).

As hypothesized, inconsistent condom users were significantly more likely to be HIV-positive (OR 1.58, 95% CI 1.10–2.26). Although this finding supports previous research on the effectiveness of consistent condom use to prevent HIV infection (*33*), our cross-sectional design renders it impossible to assess whether or not HIV was acquired when condom use consistency was assessed. Also possible is that the relationship operates in the opposite direction, i.e., that HIV seropositivity influences condom use consistency among persons aware of their status. Given, however, that consistent condom use is protective against HIV, the fact that fewer than one third of women reported consistent condom use indicates that most are at risk for future infection.

We did not find a direct association between our measures of sexual power and HIV infection, which suggests that the primary mechanism through which sexual power exerts effects on HIV risk is condom use consistency. Nevertheless, this preliminary analysis considered a limited subset of sexual power measures. As such, we cannot be certain that we captured the scope and dimensions of sexual power that have a bearing on HIV risk in ways other than through consistent condom use. Recent research conducted among antenatal clinic attendees who accepted routine HIV testing in Soweto adapted and validated the SRPS, including 12 items, for use in that context. Measured in this way, sexual relationship power was found to be associated with prevalent HIV infection (OR 1.53, 95% CI 1.10–2.04) (*34*); however, the authors did not control for condom use in their analysis, which may account for their findings. Associations between power and sexual behavior are likely to depend on sample characteristics, the conceptualization and measurement of power and risk behaviors, or a combination of these factors (*35*). Our nationally representative sample included young women from multiple regions, races, and cultures, among which key elements of sexual power dynamics are likely to differ.

The inherent limitations of our cross-sectional study design and the fact that we measured HIV prevalence, rather than incidence, may help explain the lack of an association between sexual power and HIV infection. The measures of sexual power described here refer to recent events in a current partnership, while infection may have been acquired in a prior partnership or under a different dynamic in the current partnership. We attempted to correct for this limitation by conducting a subanalysis among women 15–19 years of age with only one lifetime sex partner, who would likely have acquired HIV in the current partnership. Relationship control and HIV infection remained unassociated in this subanalysis (OR 0.98; 95% CI 0.76–1.26)2. Women who reported that their most recent partner forced them to have sex were at increased risk of HIV infection, but this association was not significant (OR 1.44; 95% CI 0.33–6.34).

Woman's sexual negotiating power is likely to be compromised in transactional sexual relationships, in relationships with older partners, and following early coital debut (*28*), and these factors would be expected to influence both condom use consistency and HIV risk. In this survey, the self-reported prevalence of all three of these behaviors was low: only 1.3% of women reported that they had transactional sex with their most recent partner; 5.5% reported that their most recent partner was >10 years older; and 7.8% reported having had sex at age 14 or younger. Transactional sex and early coital debut are particularly likely to be subject to underreporting due to social desirability bias. Further, young women whose first sexual encounter is nonconsensual, which is fairly common in this context (*8*), may not define it as "coital debut." All three of these variables were associated with increased risk of HIV infection, although the associations were not significant. Transactional sex was not associated with condom use consistency in this study. Women who reported older partners and early first sexual experience were more likely to report inconsistent condom use, though this difference was not statistically significant.

The strongest risk factor for not always using condoms with the most recent sexual partner was not having talked to that partner about condom use (OR 12.91, 95% CI 5.85–28.51). Communication between partners about contraceptive use, including condoms, has been shown to be associated with consistent use in other studies (*29*). In the context of our cross-sectional study, confirming the direction of the relationship is not possible: although couples who discuss condoms may be more likely to use them, those who consistently use condoms may also be more likely to discuss them. The relationship between sexual power and partner communication should be explored further in future research.

Given the associations between sexual power and condom use consistency, more research is warranted to assess the determinants of sexual negotiating power and to test the effectiveness of gender-sensitive HIV prevention interventions. A large national HIV prevention campaign for youth in South Africa, loveLife, has incorporated gender power issues into its media campaign by addressing issues of transactional sex, older partners, and women's lack of decision-making power in relationships (www.lovelife.org.za) (Figure). The Stepping Stones package, which is used by Planned Parenthood South Africa, also aims to challenge gender norms (*32*) and was recently found to increase women's sexual power in a pilot evaluation (*36*).

**Figure:**
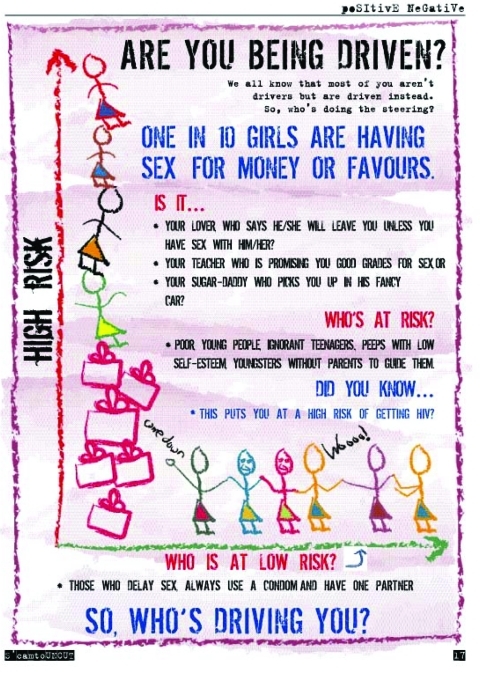
Example of message from loveLife's HIV prevention program in South Africa.

A small but growing body of research suggests that economic empowerment strategies may improve women's sexual power, with potential health benefits. In Gabarone, Botswana, economic independence was more strongly related to women's negotiating power in relationships than any other variable explored (*19*), and in Zimbabwe, adolescents who had their own income were significantly more likely to be consistent condom users (Megan Dunbar, pers. comm.). In the Limpopo province of South Africa, the Intervention with Micro-finance for AIDS and Gender Equity (IMAGE) program is being evaluated to determine its effect on gender-based violence, sexual behavior, and HIV incidence (*37*). The intervention combines a micro-finance program with a participatory learning and action curriculum. In collaboration with local partners, the University of California–San Francisco Department of Obstetrics, Gynecology, and Reproductive Sciences is currently engaged in a multisite program of research to further elucidate the linkages among economic power, sexual negotiating power, and sexually transmitted infection (STI) outcomes and to develop and test related interventions.

Debate centers around the relative effectiveness of each of the "ABCs" of HIV prevention: abstinence, being faithful to one partner, and condom use (*38*). However, all three elements likely play a role. Indeed, a decontextualized focus on these elements is likely to fail. HIV prevention strategies must take full account of the barriers persons, particularly women, face in bringing about behavior changes over which they may have little control. Many of these barriers are rooted fundamentally in gender inequalities.

## Conclusion

For a number of years, HIV activists and researchers have highlighted the role gender inequality may play in placing women at increased risk for HIV infection. At the recent International AIDS Conference in Bangkok, United Nations Secretary-General Kofi Annan made the empowerment of women and girls a priority focus area for HIV prevention: "No less pressing, empowering women and girls to protect themselves against the virus.… What is needed is positive change that will give more power and confidence to women and girls. Change that will transform relations between women and men at all levels of society." While empiric evidence documenting the relationship between women's sexual power and their HIV risk has been in short supply, a small but growing body of research confirms that women's lack of power in relationships compromises their sexual health. While this exploratory study did not find an association between sexual power and HIV serostatus, it did confirm an association between two measures of sexual power, relationship control and forced sex, and condom use consistency. Further work is needed to refine and apply measures of sexual power and to assess the complex relationship between sexual power and HIV susceptibility in the South African context. Additional research should also aim to elucidate the underpinnings of sexual power, with a particular focus on identifying avenues for intervention.
